# Setting standards for empirical bioethics research: a response to Carter and Cribb

**DOI:** 10.1186/s12910-018-0302-5

**Published:** 2018-07-13

**Authors:** Michael Dunn, Jonathan Ives, Bert Molewijk, Jan Schildmann

**Affiliations:** 1 The Ethox Centre, Nuffield Department of Population Health, Old Road Campus, University of Oxford, Headington, Oxford, OX3 7LF UK; 2 Wellcome Centre for Ethics and the Humanities, University of Oxford, Oxford, UK; 3 Centre for Ethics in Medicine, University of Bristol, Bristol, UK; 4 Department of Medical Humanities, VU Amsterdam, Amsterdam, Netherlands; 5 Institute for Medical Ethics and History of Medicine, Martin-Luther-University Halle-Wittenberg, Halle, Germany

**Keywords:** Empirical bioethics, Empirical methodology, Ethical analysis, Consensus, Delphi method

## Abstract

This paper responds to the commentaries from Stacy Carter and Alan Cribb. We pick up on two main themes in our response. First, we reflect on how the process of setting standards for empirical bioethics research entails drawing boundaries around what research counts as empirical bioethics research, and we discuss whether the standards agreed in the consensus process draw these boundaries correctly. Second, we expand on the discussion in the original paper of the role and significance of the concept of ‘integrating’ empirical methods and ethical argument as a standard for research practice within empirical bioethics.

We would like to begin by offering our thanks to Stacy Carter and Alan Cribb for their commentaries [1, 2] on the report of the process of building consensus around standards for research practice in empirical bioethics [3]. It is to their utmost credit that we found ourselves reading both sets of remarks with wry smiles on our faces. The accuracy of Carter’s and Cribb’s imagined account of how disagreement manifested itself in the consensus-building exercise was uncanny. This, we think, reflects both their understanding of the subtle but significant tensions that lie just below the surface of empirical bioethical inquiry, and their careful reading of our methodological representation of the consensus-building process.

## The contours of agreement and disagreement

We should also begin by adding that we wholeheartedly agree with the general thrust of both commentaries. We take seriously the call for this consensus-building exercise to act as a springboard to encourage continued thinking and dialogue about the enterprise of empirical bioethics. Nothing in the paper contradicts this. We also recognise that seeking to set standards risks being read as an attempt to foreclose further debate, or as the settling of important matters relating to the conduct of empirical bioethics research. We certainly do not think that matters are settled, nor did we set out with such a lofty goal in mind. Carter raises a worry that the development of standards for empirical bioethics will determine (or be read as determining) “how we should do empirical bioethics” [1]. To clarify: in no way should the paper be read as an attempt to determine the precise shape of research work that should be undertaken by empirical bioethicists. In setting out on this project, we were driven by a desire to provide more clarity about what elements of good research practice ought to undergird empirical bioethics research in order to improve quality standards and transparency within the field. Whilst we think we have achieved this more modest achievement, it is important to recognise that one of the main strengths of the standards that were agreed within our consensus process lies in the fact that they allow for a potentially unlimited range of methodological approaches and theoretical positions to be endorsed and defended. Indeed, upon reflecting on our own involvement in the process, we acknowledge that an important secondary outcome of the exercise was a new-found ability to better identify and locate the contours of disagreement that lie at the heart of the developing field of empirical bioethics.

In light of these initial remarks, our intention in this response is not to rebut, defend, or counter-argue against the points raised by either commentator. As Carter and Cribb have highlighted a number of concerns that we ourselves share, we will draw upon their remarks in a way that we hope will stimulate further conversation. We strongly believe that conversation of this kind must not end if empirical bioethics is going to continue to evolve in a carefully considered and coherent manner. Disagreements will no doubt continue, regardless of these standards, and further work is necessary to give further practical shape to the application of standards of these kinds in research activities [4]. Notwithstanding the reality of continued disagreement, we also want to pause and reflect on a core characteristic of the process. Namely, that we managed to bring together 16 diverse academics from across Europe, that this group was able to enter (and exit) the process holding diverse methodological commitments and radically different views about the nature of ethics and ethical properties, and that the group was still able to come to consensus on all the standards outlined in the original paper. Agreement was far more prevalent than disagreement, and this is no small achievement.

The commentators’ remarks cover wide-ranging territory, and we limit our response to two overarching themes that emerge from both commentaries. These are: i) the relationship between setting standards and drawing boundaries, and ii) the place of the concept of ‘integration’ as a core domain for standard-setting in empirical bioethics.

## Setting standards and drawing boundaries

As Cribb recognises, setting standards necessarily involves the drawing boundaries around research activities, and that “a necessary part of setting standards for ‘x’ is specifying what is meant by ‘x’” [2]. This is significant because specifying what is meant by empirical bioethics will have potentially problematic implications for a diverse body of researchers who self-identify as empirical bioethicists in an emerging field that strives to be as inclusive as possible. We think that these implications are inevitable. The upshot of this, as Cribb and Carter both recognise (and that participants in the consensus exercise themselves recognised), is that certain research activities that involve conducting empirical research within bioethics will not count as empirical bioethics research in the way the group determined that such research should be characterised.

What precisely is the worry here? The field of empirical bioethics, broadly construed, incorporates distinctive research activities that would be excluded from the standardised account of the aims and objectives of empirical bioethics research endorsed in the consensus process. We outlined four such activities in the original paper, but one can delineate the empirical from the ethical in ways that give shape to an even broader range of research activities e.g. [5, 6]. When we embarked on this project, we naively thought that we could make progress in obtaining agreement about *methodological*, rather than broader *research*, standards in empirical bioethics. Underlying this presumption was the idea that empirical bioethicists have a relatively clear sense of what they aim to achieve when embarking on an empirical bioethics research project, and why they aim to achieve this. Thus, what we felt was needed was a way to formally communicate and codify these aspects of our work between ourselves such that they could be recognised as having been conducted competently. As we set in place the process and recruited participants, this presumption quickly melted away. It became clear to all of us, and this was endorsed in Rounds 1–4 of consensus building, that the domains around which standards were needed included broader aspects of decision-making in research – most notably around the articulation of aims and objectives of empirical bioethics research itself.

The three standards that were agreed in the two domains of ‘Aims’ and ‘Objectives’ were debated extensively during all rounds of consensus building, and these domains do indeed function to set clear and agreed boundaries about what counts as empirical bioethics research. Notably, research of this kind should “address a normative issue that is oriented towards practice” (Standard 1), and it should “integrate empirical methods with ethical argument in order to address this normative issue” (Standard 2).

Carter draws attention to one form of research activity that we claimed would be excluded: “empirical research that reveals the form and nature of how ethical issues arise in practical situations” [1]. She also draws our attention to two other forms of research activity: developing “new insights that could broaden one’s moral horizon” and explanations of “relevant aspects of the problem” [1] that we claimed would be included. One concern with drawing boundaries in this way is that excluding the first research activity “slices off much useful work from Australia, the UK and elsewhere that explains the moral reasoning and practices of lay people and professionals” [2]. The implicit point being made here is that this work would be rendered less useful if it was excluded, or sliced off, in such a manner. There is no good reason, however, to think that merely falling outside the boundaries of standards for empirical bioethics research, as defined in the original paper, renders that work any less valuable, or of lesser quality, than work that falls within such boundaries. There could of course be very important reasons for undertaking such research, even if it is not research that would meet the requirements to be described as empirical bioethics. Standards 1, 2 and 3 are definitional in character. Failing to meet these three standards, therefore, implies nothing about the fundamental *quality* of the research activity being undertaken, just whether or not it is activity of the relevant kind.

Precisely whether particular empirical research activities fall within or outside these standards for empirical bioethics on the basis of the first three standards would benefit from additional clarification on our part. Drawing the correct boundaries here will depend fundamentally on what these research activities set out to achieve. The reason we excluded the first type of research activity mentioned by Carter was because we imagined that revealing the form and nature of how ethical issues arise in practical situations functioned as an end in itself. On this account, the aim would not be to judge how those issues ought to be addressed, or how people ought to behave, but to expose and explore the moral complexities that arise for people in the world, in their own terms. As the description and explanation of patterns of moral behaviour, attitudes, or experiences are the goals of inquiry, then this activity does not aim to address a normative issue, and would fail to meet Standard 1. This would be the case even if, as a consequence of this activity, participants in the research changed their behaviour or practice as a result of their contributions to determining the content of the ethical issues they faced. We might better characterise research of this kind as ‘descriptive ethics’, even if it has non-intended normative consequences.

In contrast, activities that involve ‘expanding one’s moral horizon’ appear, at least at first glance, to be normative activities, when this phrase is taken to capture research that determines how identified ethical considerations *should* be taken into account. Equally, there might well be ways of describing a study where the phrase “expand one’s moral horizon” is drawn upon such that the empirical research conducted is not focused on addressing a normative issue. The key question, according to the requirement of Standard 1 and regardless of how the research activity is named, is: *does the empirical research* – taking place in a single study or as a component of a broader programme of research activities – *aim to address a normative issue*?

We recognise that this response might not satisfy all of our readers. Some disagreement will revolve around the phrases we have used to describe certain research activities, which imply descriptive or normative orientations that are not as clear as they perhaps could be. It is likely that any residual disagreement is likely to concern how the distinction between empirical and normative research aims trades off background disagreements within the social sciences about the normative content of empirical research. This is particularly an issue in those empirical (especially qualitative) traditions that are derived from social theoretical positions that are founded on strong normative presuppositions [7]. In such empirical research, the empirical activity itself is likely to look very similar to empirical bioethics research, and will involve making (implicitly or explicitly) normative claims as part of the discussion of the empirical findings. Thus, in Marxist or feminist traditions in the social sciences, for example, it is very likely that qualitative empirical research methodologies that are derived from the main tenets of the theories within these traditions will qualify as empirical bioethics research under Standard 1. There will be additional questions about whether such research meets Standard 2, or those standards specified within Domain 5, and this will depend on whether ethical argument is integrated with empirical methods in the research activity, or if it is an additional project component in a wider programme of bioethics research. Quite what is required by reference to integration here is where we now turn.

## The requirement to integrate empirical research with ethical argument

Both Carter and Cribb discuss the central place given to the requirement to ‘integrate’ empirical research and ethical argument in the agreed standards. A number of concerns are expressed, with Carter drawing attention to more conceptual objections, and Cribb outlining some more practical worries. One of Carter’s concerns is that the concept of integration, as articulated through the relevant standards, assumes the separability of the descriptive and the normative. A second concern is that the process of determining the rightful place of integration in standard-setting was circular in character. This, she claims, is because participants were asked to accept this concept when signing up for their involvement in consensus-building, thus assuming and ‘writing-in’ separability throughout the entire process.

On the second charge first. It is certainly true that we have drawn extensively on the concept of integration as a defining feature of the attempt to differentiate empirical bioethics research from other ways of making use of empirical research in bioethics (Ives et al., book, intro). This goes some way to explain why we included reference to integration in describing ‘a minimal account of empirical bioethics’ at the start of the process. Rounds 1 and 2, however, involved considerable debate about the domains within which standards needed to be agreed. As we discussed above, it was within this phase of the process that we abandoned the minimal account of empirical bioethics that had been circulated prior to the meeting, and recognised that separate ‘definitional’ domains of Aims and Objectives needed to be considered in the standard-setting process. This led to the concept of integration being extensively debated, and a recognition that specific standards associated with the requirement to integrate empirical research with ethical argument were necessary. Thus, participants spent considerable time debating the appropriateness, and rightful place, of the concept of integration in empirical bioethics. Parallel discussions led to a number of other domains, standards, and concepts being abandoned or reworked. The concept of integration survived this process. Here, we rely on the robustness of the consensus-building process we adopted, and believe that the activities that constituted this process provide a compelling response to any concerns about circularity.

But what of the problematic broader assumptions that underlie the concept of integration as endorsed in the project? One unfortunate connotation of this word is that it is often taken to refer to a procedural or methodological requirement to connect different (disciplinary) research activities. The consensus process did not signify the endorsement of this interpretation. We recognise that this term will be uncomfortable, awkward, or misplaced for some empirical bioethicists, but, in the process, this term was preferred to alternatives like ‘connection’, ‘relation’ or ‘co-existence’ (a term which Cribb favours in his commentary), which were considered and ultimately rejected. The consensus view was taken that those empirical bioethics researchers who believe that the empirical and the normative are inseparable will need to carefully describe why integration is not directly relevant to them, and precisely how the empirical and normative relate in their work in ways that avoid a specific need to integrate different research activities within their methodological design.

Once this has been done, these researchers will also need to carefully consider how they meet standards within Domains 4 and 5 (‘Conduct of empirical work’ and ‘Conduct of normative work’), in light of their view about the precise relationship between the empirical and the normative within Domain 3. Denial of the fact-value distinction is likely to favour more entangled methodologies, and its acceptance is likely to favour distinctive empirical and ethical phases in an empirical bioethics project, but this need not be the case. It is entirely coherent to hold the view that facts and values are inseparable, but still to endorse a separation of empirical and normative analytic phases within the design of an empirical bioethics research project. Equally, it also coherent to endorse the fact-value distinction but also to favour an approach to empirical bioethics research that interweaves empirical and normative analysis in complex methodological designs.

Cribb claims that one upshot of these standards is that “different strands of research within a large research programme, and sometimes within a single project, will fall either side of this boundary [of what counts as empirical bioethics]” [3]. Cribb is right to draw attention to the practical difficulty that will arise in how the standards are applied in project management terms. An important way in which the concept of integration is formulated means that it could apply at different scales of research activity: from sub-project to overarching programme. This is an issue for empirical bioethics research in just the same way as it is for bioethics research more generally – where a justified answer to a primary, normative bioethics research question is likely to allow for a number of detailed research objectives, and a wide range of multi-disciplinary methodological strategies, within an overarching bioethics research project, that intersect in complex ways [8]. Thus, integration could happen across an entire research programme, when empirical and ethical analytic methodologies are adopted in phased research project activities, or a single empirical ethics project might involve a discrete piece of research where empirical analysis and ethical argument are fully intertwined. Which approach is to be preferred will, in large part, depend on articulating a research design that adheres to the kinds of standards put forward in the original paper. We hope that readers will approach these standards in the constructive way that they are intended to be engaged with. Strong disagreement with what was agreed in the consensus-building process could be just as productive for the further evolution of empirical bioethics research as widespread agreement will be, and so should not be shunned. We look forward to more exchanges on how to further develop and refine these standards as debates on the nature and quality of empirical bioethics continue into the future.
